# Massive Esophageal Hemorrhage From a Large Esophageal Tear Associated With Chronic Gastroesophageal Junction Stenosis Managed With Dual Overlapping Covered WallFlex Stents

**DOI:** 10.7759/cureus.110924

**Published:** 2026-06-15

**Authors:** Noah Lubin, Priya Vijayvargiya, Arslan Kahloon, Laurie-Anne Swaby

**Affiliations:** 1 Internal Medicine, University of Tennessee Health Science Center College of Medicine, Chattanooga, USA; 2 Gastroenterology, University of Tennessee Health Science Center College of Medicine, Chattanooga, USA

**Keywords:** esophageal hemorrhage, esophageal tear, gastroesophageal junction stenosis, ge junction stenosis, schatzki ring, self-expanding metal stents (sems)

## Abstract

Recurrent gastroesophageal (GE) junction stenosis and a Schatzki ring can rarely lead to severe esophageal injury and life-threatening bleeding. We describe a 68-year-old patient on apixaban with longstanding dysphagia requiring numerous prior esophageal dilations who presented with hematemesis and hemorrhagic shock from a large esophageal tear associated with pill impaction and severe distal esophageal narrowing. Initial endoscopic and radiologic interventions were unsuccessful in controlling the bleeding. Repeat endoscopy identified an extensive mucosal tear with ongoing hemorrhage, which was successfully managed with placement of dual overlapping fully covered esophageal stents under fluoroscopic guidance, spanning the injured segment and stenotic area. The patient recovered without recurrent bleeding and later underwent planned stent removal. This case highlights the potential complications of recurrent esophageal dilation and demonstrates the possible role of covered esophageal stents in the management of refractory non-variceal esophageal bleeding.

## Introduction

Schatzki rings are sharply localized esophageal lesions with clearly defined margins that are a well-known cause of intermittent solid food dysphagia; they are present in 15%-18% of patients undergoing esophagram for the evaluation of dysphagia [1,2]. Although some patients with Schatzki rings have improvement in symptoms following one esophageal dilation, thicker Schatzki rings with dense fibrotic tissue are more likely to require repeat esophageal dilation [1]. Esophageal dilations are generally safe, with reported perforation rates between 0.09% and 2.2% [3]; rates of esophageal rupture are 1.7% for bougie dilation and 3% for small balloon dilation [4]. This perforation risk increases in patients with complex strictures or those requiring repeated interventions [5,6].

Covered self-expanding metal stents (SEMS) have established roles in managing esophageal perforations, anastomotic leaks, and refractory variceal bleeding [3,7,8]. However, the literature remains sparse regarding dual overlapping stents in the treatment of non-variceal esophageal bleeding, particularly in the setting of large mucosal tears [9]. Traditional management of non-variceal esophageal bleeding is frequently achieved with endoscopic hemostasis techniques, with interventional radiology (IR) embolization reserved for refractory cases [10,11]. When these conventional measures are not sufficient to control massive non-variceal esophageal bleeding, SEMS can achieve hemostasis through mechanical compression of the bleeding site rather than direct treatment of the underlying lesion.

We present a rare case of hemorrhagic shock secondary to a large esophageal tear from suspected pill impaction in the setting of severe gastroesophageal (GE) junction stenosis in a patient with over 22 prior esophageal dilations; the bleeding was successfully managed with dual overlapping fully covered WallFlex stents. This case highlights both the potential complications of recurrent esophageal dilations and the utility of covered metal stents as a bridge therapy for non-variceal esophageal bleeding.

## Case presentation

A 68-year-old man with a medical history significant for atrial fibrillation on apixaban, hiatal hernia, and recurrent dysphagia due to GE junction stenosis from a Schatzki ring presented to an outside hospital with progressive dysphagia and hematemesis after taking one ibuprofen pill. He notably had required more than 22 prior esophageal dilations for dysphagia over the previous several years, performed every 2-3 months with either a balloon or bougie dilator and without prior complication; his most recent dilation was 3 months before presentation. Initial esophagogastroduodenoscopy (EGD) at presentation was limited by extensive clot burden, and no definitive bleeding source could be identified. The patient received prothrombin complex concentrate (PCC) for reversal of apixaban. Computed tomography angiography demonstrated active mid-esophageal extravasation (Figure 1). IR was consulted for embolization but failed to identify a suitable target vessel. Given the ongoing bleeding and hemodynamic instability, the patient was transferred to our tertiary care center for higher-level management.

**Figure 1:**
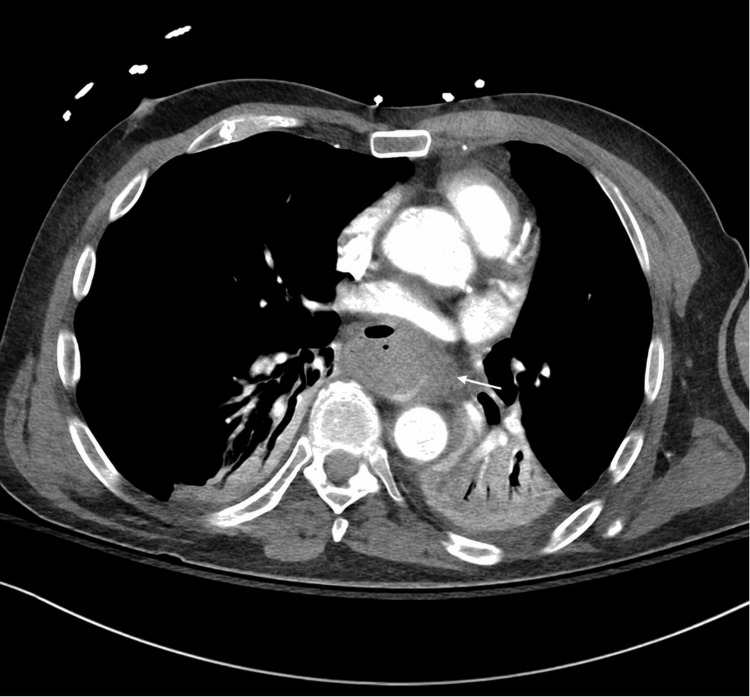
Computed tomography angiography (CTA) of the chest. White arrow: Active contrast extravasation in the posterior aspect of the lower thoracic esophagus.

On arrival, the patient had ongoing hematemesis and required low-dose norepinephrine for hemodynamic support; his hemoglobin decreased from 10.4 g/dL to 8.4 g/dL over several hours. He ultimately required transfusion of 5 units of packed RBCs. Repeat EGD revealed massive clot burden throughout the esophagus, a cratered distal esophageal ulcer with two visible vessels, and severe GE junction stenosis. The stenosis was carefully traversed using a neonatal endoscope, revealing a deep mucosal tear extending from 28 cm to 43 cm from the incisors. The tear bled briskly with clot displacement, confirming it as the primary source of hemorrhage (Figure 2A).

**Figure 2:**
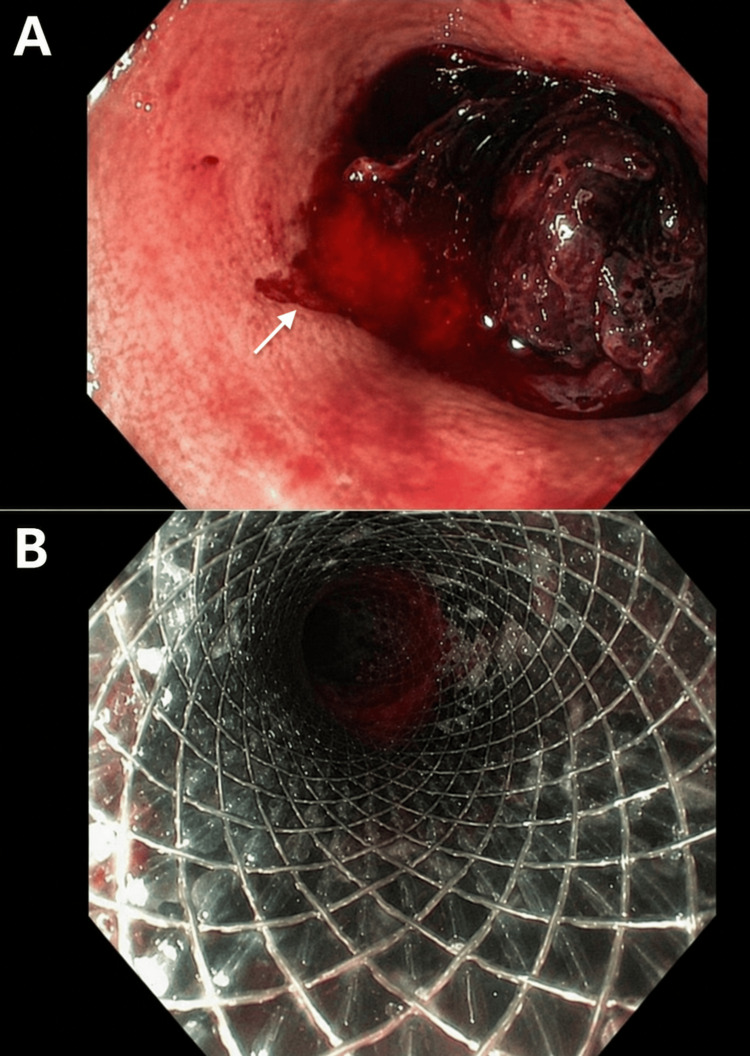
Esophagogastroduodenoscopy (EGD) images. A: Esophageal clot burden, longitudinal mucosal tear, and active bleeding. White arrow: Edge of the mucosal tear. B: Deployment of a self-expanding metal stent (SEMS) across the gastroesophageal (GE) junction.

Given the extensive length of the esophageal tear (15 cm) and ongoing bleeding despite attempted endoscopic hemostasis with epinephrine injection, the decision was made to deploy dual overlapping covered SEMS. Two fully covered WallFlex SEMS (18 mm × 15.3 cm stent, followed by 18 mm × 10.3 cm stent) were placed under fluoroscopic guidance to span the entire length of the esophageal tear and GE junction stenosis (Figures 3-4).

**Figure 3:**
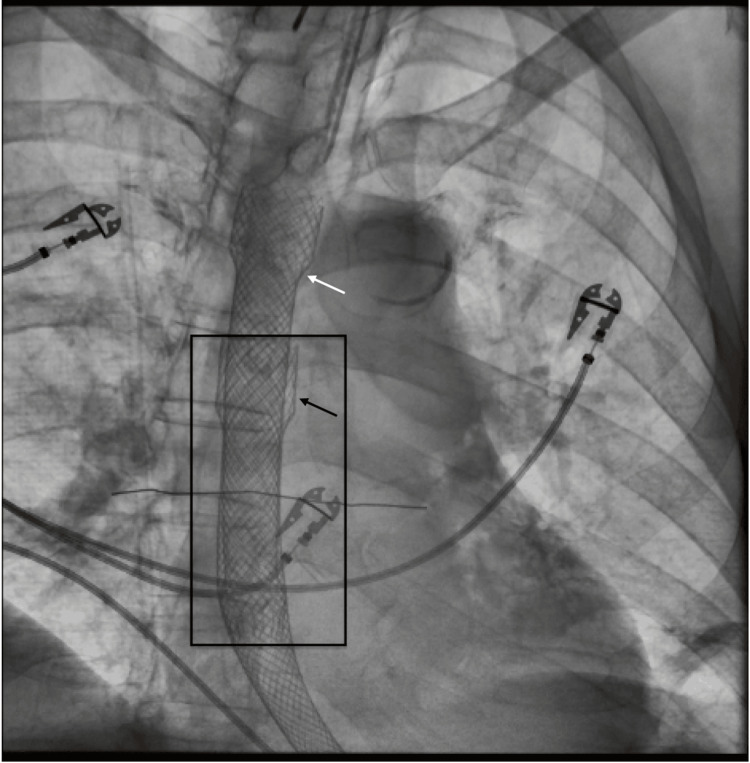
Fluoroscopic localization of esophageal stents, upper chest view. White arrow: First WallFlex stent. Black arrow: Second WallFlex stent. Black box: Area of overlap between the WallFlex stents.

**Figure 4:**
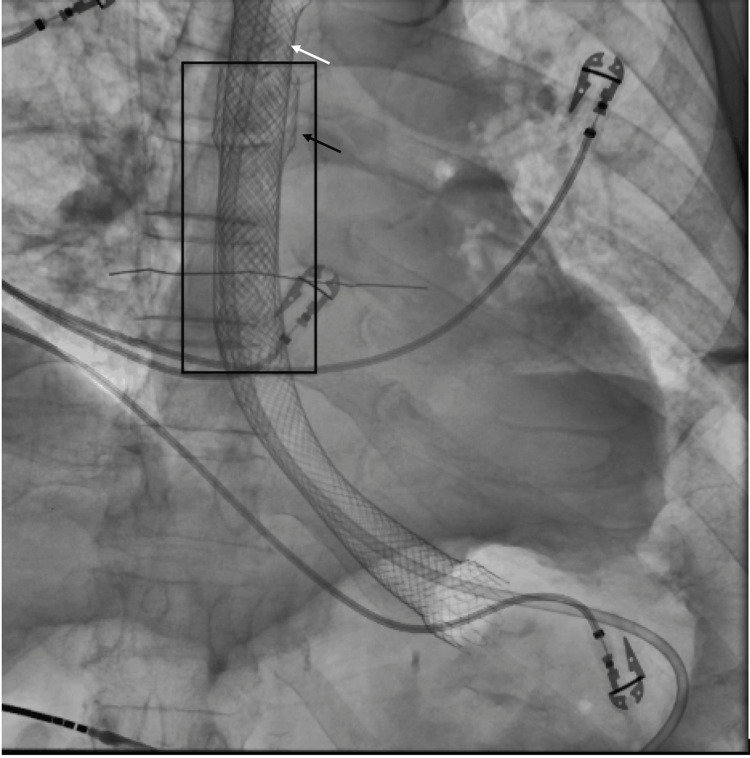
Fluoroscopic localization of esophageal stents, lower chest view. White arrow: First WallFlex stent. Black arrow: Second WallFlex stent. Black box: Area of overlap between the WallFlex stents.

Following stent placement, the patient's hemoglobin increased to 11.7 g/dL, and he showed no further evidence of overt bleeding. He was successfully extubated the following day and tolerated oral intake without difficulty. Apixaban was cautiously resumed 6 days after stent placement without further bleeding episodes. The patient was discharged the next day (post-procedure day 7) with plans for repeat endoscopy and stent removal. Follow-up endoscopy was completed one month later, with removal of both stents. EGD at that time showed congested, erythematous mucosa in the esophagus and Los Angeles Grade D esophagitis, but his stenosis had improved. There was no further documentation of dysphagia or need for further dilation in the five months following stent removal.

## Discussion

This case illustrates several important clinical considerations in the management of complex benign esophageal disease. First, it highlights the potential complications associated with recurrent esophageal dilations. While current guidelines support repeat dilations for recurrent strictures with no reported limit to the number of dilation sessions, this patient's history of over 22 dilations raises questions about when alternative management strategies should be considered [12]. Notably, his esophageal tear was thought to be due to pill impaction in the setting of esophageal wall fragility from multiple dilations and chronic GE junction stenosis.

The American Society for Gastrointestinal Endoscopy defines refractory strictures as those that cannot be successfully dilated to 14 mm over 5 sessions at 2-week intervals or those that cannot maintain a satisfactory luminal diameter for 4 weeks once the target diameter is achieved [12]. A recent study showed that 47.1% of patients with Schatzki rings required repeat dilations [1]. Risk factors for perforation during dilation include male sex, age ≥70 years, distal esophageal location, smaller initial stricture diameter, and the presence of complex strictures [5]; this patient possessed several of these risk factors.

The role and optimal timing of definitive alternative management, including jumbo cold biopsy forceps removal, electrosurgical incision, or Nissen fundoplication for associated hiatal hernia, in patients with recurrent fibrosed Schatzki rings and hiatal hernia deserve consideration. While no specific guidelines exist limiting the number of dilations before considering surgery, this patient's course suggests that the aforementioned alternative approaches should be strongly considered after more than 20 procedures with incomplete symptom resolution. Some factors that may favor surgical intervention in these patients include young age, good functional status, and the presence of a large hiatal hernia, which dilation does not address. Esophagectomy represents a last resort for benign disease when the esophagus has become non-functional and significantly impacts quality of life [13].

The use of covered esophageal stents for non-variceal bleeding represents an innovative application of this technology. Zhou Y et al. reported a case series of four patients with refractory esophageal non-variceal bleeding successfully treated with covered SEMS, with immediate hemostasis achieved in all cases [9]. The mechanism of action involves direct compression of the bleeding vessel and creation of a barrier preventing further hemorrhage. In our case, the dual overlapping stent configuration was necessary to span the 15 cm tear, ensuring complete coverage and adequate hemostatic pressure along the entire length of the injury. Temporary stent placement across the area of stenosis also allowed for continuous luminal expansion and remodeling of scar tissue to prevent recurrence of stricture. Further studies are needed to clarify the risks of stent migration, tissue overgrowth, and pressure injury in dual overlapping stents compared with single stent placement.

Typically, significant gastrointestinal bleeds that fail endoscopic management can undergo embolization with IR. However, this is challenging for esophageal bleeds secondary to the multiple branches supplying the esophagus, including the inferior thyroid artery, bronchial arteries, and left gastric artery. Embolization of esophageal vessels carries significant risk of ischemia and perforation, and prophylactic embolization without visualized extravasation is generally not recommended [14].

## Conclusions

This case demonstrates that massive esophageal hemorrhage from large mucosal tears can occur as a rare but serious complication of chronic GE junction stenosis. Covered SEMS represent a valuable option for managing refractory non-variceal esophageal bleeding when conventional endoscopic hemostatic techniques fail and IR embolization is not feasible. The successful use of dual overlapping WallFlex stents in this case highlights their utility in achieving hemostasis and facilitating subsequent management.

This case also emphasizes the challenges of long-term management in patients with refractory benign esophageal strictures requiring repeated dilations. Earlier consideration of alternative definitive treatment strategies may be warranted when symptom control remains inadequate despite serial endoscopic therapy. Further studies are needed to better define optimal management pathways and criteria for escalation of care in patients with recurrent benign esophageal strictures.
